# Development of Solar Drying Model for Selected Cambodian Fish Species

**DOI:** 10.1155/2014/439431

**Published:** 2014-08-27

**Authors:** Anna Hubackova, Iva Kucerova, Rithy Chrun, Petra Chaloupkova, Jan Banout

**Affiliations:** ^1^Department of Sustainable Technologies, Faculty of Tropical AgriSciences, Czech University of Life Sciences Prague, Kamycka 129, Suchdol, 16521 Praha 6, Czech Republic; ^2^Department of Food Biotechnology, Faculty of Agro-Industry, Royal University of Agriculture, P.O. Box 2696, Khan Dangkor, Phnom Penh, Cambodia; ^3^Department of Economics and Development, Faculty of Tropical AgriSciences, Czech University of Life Sciences Prague, Kamycka 129, Suchdol, 16521 Praha 6, Czech Republic

## Abstract

A solar drying was investigated as one of perspective techniques for fish processing in Cambodia. The solar drying was compared to conventional drying in electric oven. Five typical Cambodian fish species were selected for this study. Mean solar drying temperature and drying air relative humidity were 55.6°C and 19.9%, respectively. The overall solar dryer efficiency was 12.37%, which is typical for natural convection solar dryers. An average evaporative capacity of solar dryer was 0.049 kg*·*h^−1^. Based on coefficient of determination (*R* ^2^), chi-square (*χ* ^2^) test, and root-mean-square error (RMSE), the most suitable models describing natural convection solar drying kinetics were Logarithmic model, Diffusion approximate model, and Two-term model for climbing perch and Nile tilapia, swamp eel and walking catfish and Channa fish, respectively. In case of electric oven drying, the Modified Page 1 model shows the best results for all investigated fish species except Channa fish where the two-term model is the best one. Sensory evaluation shows that most preferable fish is climbing perch, followed by Nile tilapia and walking catfish. This study brings new knowledge about drying kinetics of fresh water fish species in Cambodia and confirms the solar drying as acceptable technology for fish processing.

## 1. Introduction

Despite continued technological development, advances in information technology, and ever increasing globalization, a great part of the population in developing countries suffers from lack of access to electricity. Cambodia is an example of a country where only 34% of population had access to electricity [1]. At the same time, more than 85% of the population in Cambodia is strongly dependent on agriculture, from which freshwater aquaculture is one of most important sources of food production [2, 3]. In 2009, over 420 000 of people were directly employed in the fisheries sector, accounting for almost 5% of the Cambodian workforce. Furthermore, it is estimated that the livelihood of more than 2 million people depends in some way on this sector [3].

Fresh fish meat contain up to 80% of water by mass and it is considered as highly perishable material, which results in an extremely short shelf-life when left unprocessed [4]. Since preservation enables storage and transport and thus opens up the possibility of trade, proper preservation techniques are significant not only for ensuring the local food supply but may stimulate economic development in a wider region. The benefits to farmers themselves are in allowing them to maintain a constant price of their products, improving their bargaining position and widening their possible market [5–7]. Many preservation techniques such as fermenting, smoking, frying, salting, and conversion into fish sauce or paste have been developed. Solar drying is one of the most attractive and promising solar energy systems, as it is simple, does not require much initial investment, and can be very effective, especially in tropical regions [5]. Preservation techniques in general depend on processes that lower water activity of the preserved food (*a*_*W*_) and thus inhibit or prevent the activity of undesirable microorganisms and enzymes that require aqueous environment, as well as the growth of mold and fungi [8]. In drying, this is achieved by actively removing water itself from the food matrix [5].

Since there is only limited access to electricity and other energy resources in rural Cambodian communities, most of the local fish production is processed using only the most basic preservation method, which is open sun drying. While it is the most easily accessible means of preservation, open sun drying has major disadvantages. First, it requires a large open space area exposed to direct sunlight. Second, it is generally inefficient. The fish are often dried to an unstable moisture content, which is conducive to microorganism proliferation, and consuming such food may lead to food poisoning [5, 9]. Third, open sun drying exposes the dried food to dust, bird excrements, or insect and rodent infestation and as such it is highly unhygienic [9, 10]. Apart from the simple open sun drying method, there are certain more advanced drying methods that make use of solar energy. The solar drying system is a significantly more hygienic and effective alternative to open air drying, although it is still affordable and simple [11]. There are several classes of dryers: natural and forced convection solar dryers, direct solar dryers [12–15], and indirect solar dryers [16–21]. Many studies from Asia are specifically focused on processing of plant products by drying [5, 22, 23], but less data are available for meat and fish drying. Thus, the purpose of this study is the evaluation of solar drying of five common Cambodian fish species as an alternative to traditionally used open sun drying and/or conventional dryers supplied by electric power. The evaluation of mathematical models for thin layer solar drying of fish as well as the influence of drying method on organoleptic properties of dried fish were investigated during this study.

## 2. Materials and Methods

### 2.1. Material

Based on local survey conducted in biggest fish markets in Phnom Penh, five common Cambodian fish species, namely, swamp eel (*Monopterus albus*), Nile tilapia (*Oreochromis niloticus*), walking catfish (*Clarias batrachus*), Channa (*Channa lucius*), and climbing perch (*Anabas testudineus*) were selected for this study as locally most typical and most frequently marketed. Samples of above mentioned fish species were purchased at the local market near the Royal University of Agriculture (RUA) in Phnom Penh, Cambodia. The fish were cleaned and sliced into pieces of approximately 5 by 2 cm. The fish were seasoned according to local recipes (combination of salt, black pepper, chili, garlic, ginger, lemon, and lemongrass). The samples were then placed into one of two types of dryers, the electric oven (EO) used as the control and the solar dryer (SD).

### 2.2. Drying Facilities

**Figure 1:**
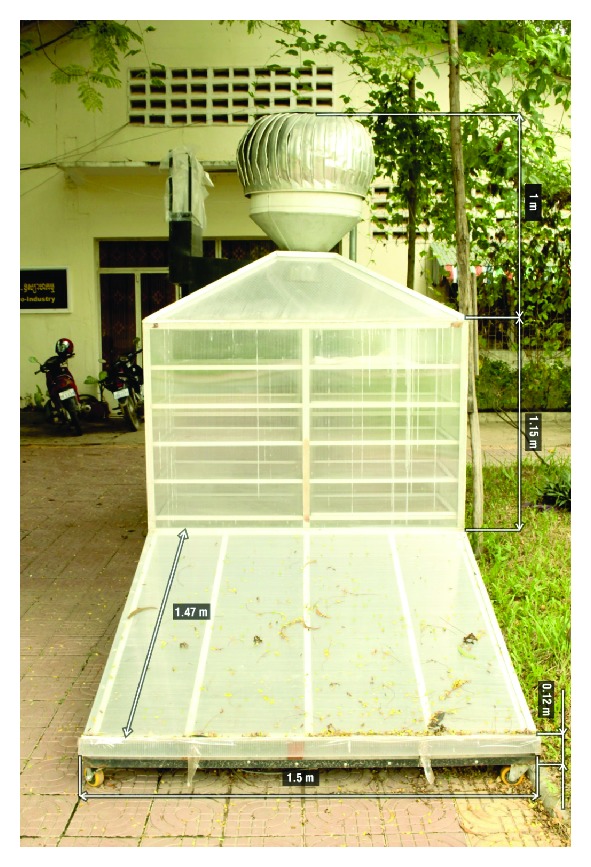
The schematic picture of natural convection solar dryer.

The control drying was done in electric oven (UFE 500 type, Memmert, Germany) with stable temperature 60°C and air relative humidity 16.2%. The solar drying was conducted in SD installed in the campus of RUA in Phnom Penh, 2013. The drying system was classified to be of the natural convection direct type. A picture of the solar dryer is shown in Figure 1. The solar dryer consisted of a solar air heater collector, drying chamber with drying trays and a blower, connected to the top of the drying chamber. The collector width, length, and depth were 1.50 m, 1.47 m, and 0.12 m, respectively. The solar collector array consists of a solid transparent plastic cover, an insulator, and a black painted aluminum absorber. Air enters into the drying chamber trough the collector by natural convection mode. The chamber dimensions are 1.50 m long, 0.60 m wide, and 1.10 m tall.

### 2.3. Instrumentation and Experimental Procedure

Solar drying experiments started at 1:00 PM during the first day of drying and at 8:00 AM during the next two days. The drying was stopped always at 5:00 PM during all drying tests. In the night, the samples were collected and placed to the room in closed plastic boxes. During the drying process, moisture loss was monitored at hourly intervals using a digital weighing scale (Soehnle Professional, Backnang, Germany) with a 0.1 g precision uncertainty. Except for the moisture loss, additional operational parameters were monitored at hourly intervals. Ambient and drying air relative humidity and temperature were measured by Minidataloggers Testo 174H (Testo, Lenzkirch, Germany) installed outside the solar dryer and in the drying chamber. Insolation on the collectors of the dryer was measured by pyranometer CMP 6 with a solar integrator (KippZonen, Delft, the Netherlands) with daily accuracy ±5%. Anemometer Testo 425 (Testo, Lenzkirch, Germany) with an accuracy ±0.03 m*·*s^−1^ was used to determine the air velocity. At the end of the drying tests, samples of each fish were placed in the electric oven for 24 h at 105°C for determination of dry matter content.

### 2.4. Performance of Solar Dryer

To evaluate drying performance of solar dryer, thermal efficiency and system drying efficiency (*η*_*p*_) were calculated from the data obtained during the drying experiments. An equation of the thermal efficiency of a solar collector (*η*_*c*_) is the ratio of useful heat gain the solar radiation acting on the solar collector and can be calculated as follows [24]:

(1) $$\begin{array}{c} \eta_{c}=\frac{M\cdot C\left(T_{0}-T_{i}\right)}{A_{C}\cdot I}\times 100. \end{array}$$

The system drying efficiency (*η*_*p*_) describing how effectively the input energy to the drying system is used in product drying. For collector type natural convection solar dryers, the heat supplied to the dryer is the solar radiation incident on the plane of solar collector and may be expressed as follows [25]:

(2) $$\begin{array}{c} \eta_{p}=\frac{W\cdot L}{A_{C}\cdot I}\times 100. \end{array}$$

The quantity of moisture evaporated from the dried material could be calculated as mass of water evaporated from the product (*W*) and presented by the following equation [24]:

(3) $$\begin{array}{c} W=\frac{m_{0}\left(M_{i}-M_{f}\right)}{100-M_{f}}. \end{array}$$

Drying rate is a fundamental parameter in the evaluation of drying process. Kituu et al. [11] evaluated the drying rate (DR) as the decrease of the water concentration during the time interval between two subsequent measurements divided by time interval. Drying rate (DR) could be expressed as

(4) $$\begin{array}{c} \text{DR}=\frac{\Delta M}{\Delta T}. \end{array}$$

Jannot and Coulibaly [26] established evaporative capacity that is the measure of the effect of air temperature and humidity. It could be expressed as weight of water that can be extracted by the air flow from the product to be dried:

(5) $$\begin{array}{c} E=m\left(X_{2m}-X_{a}\right). \end{array}$$

### 2.5. Mathematical Modeling of Drying Curves

Fick's diffusion equation for solid materials with slab geometry was applied to the experimental data during fish drying. The assumption made for the slab shape of dried sliced fish samples was that moisture is initially uniformly distributed throughout the mass of a sample. Surface moisture content of the sample instantaneously reaches equilibrium with the condition of surrounding air. Resistance to mass transfer at the surface is negligible compared to internal resistance of the sample. The equation is as presented below [27]:

(6) $$\begin{array}{c} \text{MR}=\frac{M-M_{e}}{M_{i}-M_{e}}=\frac{8}{\pi^{2}}\exp\left(\frac{-\pi^{2}D_{\text{eff}}t}{4L^{2}}\right). \end{array}$$

The drying data were graphically analyzed in terms of reduction in moisture content and moisture ratio with drying time. The moisture ratio MR expressed in (7) was taken instead of the moisture ratio presented in (8) [28]:

(7) MR=M−MeMi−Me,

(8) MR=MM0.

The reason of this simplification was that, in the solar drying, the relative humidity of the drying air continuously fluctuated. The solar drying curves were fitted with ten different moisture ratio equations [29–31] presented in Table 1.

The coefficient of determination (*R* ^2^) was used as one of the primary criterion for selecting the best mathematical model describing the solar drying curve of fish samples. In addition to *R* ^2^, chi-square (*χ* ^2^) and root-mean-square error (RMSE) were used to analyze the relative goodness of fit. The model with the highest coefficient of determination and the lowest *χ* ^2^ and RMSE was selected as the best model describing the drying behavior of fish. Coefficient of determination and chi-square are defined by [32]

(9) R2=1−(∑i=1N(MRexp⁡,i−MRpre,i)2∑i=1N(M− R−exp⁡,i−MRpre,i)2),χ2=∑i=1N(MRexp⁡,i−MRpre,i)2N−z.

Root-mean-square error is expressed by [33]

(10) $$\begin{array}{c} \text{RMSE}={\left[\frac{1}{N}\overset{N}{\underset{i=1}{\sum }}{\left({\text{MR}}_{\exp,i}-{\text{MR}}_{\text{pre},i}\right)}^{2}\right]}^{1/2}. \end{array}$$

### 2.6. Organoleptic Properties and Sensory Analysis

Organoleptic properties and sensory analysis of samples of dried fish were conducted by 19 trained panelists. The facility used for the sensory evaluation was a large room and each panelist was supplied with questionnaire, a pencil, a glass of water, and all the panelists were allowed into the room together and had unlimited time to complete the testing. Following criteria were judged during the analysis: appearance, odour, flavor, texture, and overall sensory quality. Each sample of fish was evaluated for overall acceptability using a five point hedonic scale (1—excellent, 5—poor). Data was analyzed using statistical method ANOVA on 5% significance level in statistical program Statistica software version 10.0 (StatSoft Inc., Oklahoma, USA). Fisher's LSD test was used to determine which fish samples differ from others.

## 3. Results and Discussion

### 3.1. Dryer Performance

**Figure 2:**
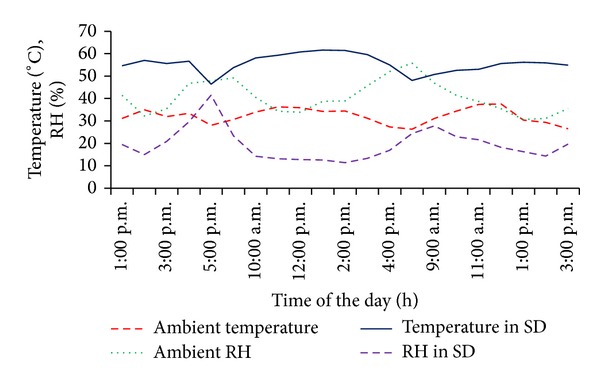
Air temperatures, air relative humidity, and solar radiation patterns during typical drying experiment.

All drying conditions of the solar drying process were monitored and they are presented in Figure 2. The values of ambient temperature, ambient relative humidity (RH), and solar radiation ranged between 26.3°C and 37.6°C, 30.6% and 55.8%, and 236.2 W*·*m^−2^ and 873.4 W*·*m^−2^, respectively. Temperature and relative humidity of the drying air ranged between 46.4°C and 61.4°C and 11.4% and 29.6%. From the curves presented in Figure 2, it is clear that drying air temperature and drying air relative humidity have a contradictory run. Moreover, it is evident that the maximum drying temperatures between 11:00 AM and 2:00 PM did not exceed 70°C which is considered as a maximum temperature for fish drying [34]. It was observed that the mean drying temperature and drying air relative humidity in the solar dryer were in average about 72.48% higher and 51.96% lower than the ambient ones.

**Figure 3:**
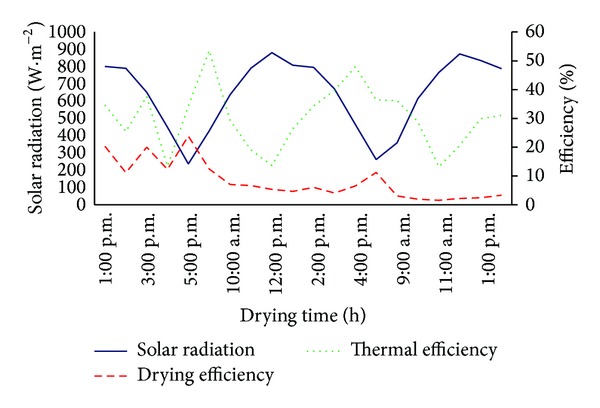
Thermal efficiency and drying efficiency as compared to solar radiation for typical experiment.

A performance of solar dryer was calculated according to (1), (2), and (3). The overall drying efficiency and thermal efficiency varied during whole drying process from 1.56% to 23.85% and from 13.16% to 53.56%, respectively.  Figure 3 shows that minimal solar radiation corresponds to highest drying and thermal efficiency. Similar observations were reported by Fudholi et al. [24]. Further, the overall average dryer efficiency was 12.37%. This value corresponds to the desired safe moisture content of dried fish meat which is equal to 15% [4]. Obtained drying efficiency representing the upper limit of efficiencies from 10% to 15% which are typical for natural convection dryers [35].

**Figure 4:**
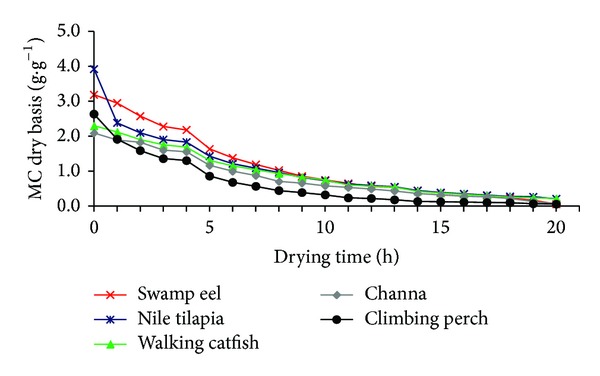
Changes of moisture content (db) of different fish meat samples with drying time for a typical experimental run in solar dryer (SD).

**Figure 5:**
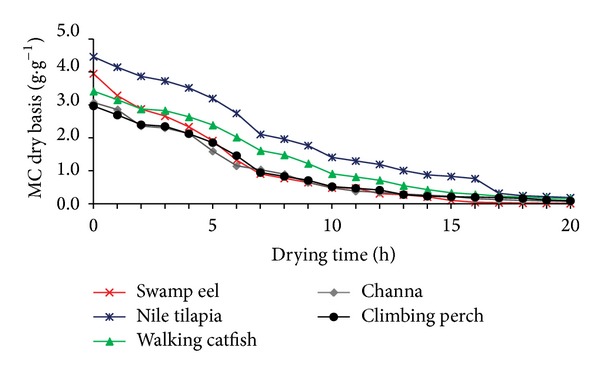
Changes of moisture content (db) of different fish meat samples with drying time for a typical experimental run in electric oven (EO).

Evaporative capacity helps to evaluate the influence of meteorological conditions on solar dryer performance. In some cases the evaporative capacity is more precise index to evaluate the solar dryer performance as compared to traditionally used thermal efficiency, especially when particular use with the preheated air is considered. An average evaporative capacity of solar dryer was calculated using (5) and it was equal to 0.049 kg*·*h^−1^. The evaporative capacity increased with increasing solar radiation. The average initial moisture content (MC) of fish meat from all species varied between 73.12% and 77.82% (wb). Figures 4 and 5 present the reduction of moisture content with time in SD and EO. After 20 hours of drying, the final moisture content decreased to 3.13% and 2.22% (wb) in solar dryer and electric oven, respectively.

From Figures 4 and 5, it is evident that, in general, a higher drying rate was achieved in SD as compared to EO. This fact corresponds to higher drying air temperature and lower RH during solar drying. Focusing on the drying curves of different fish species dried under constant temperature in EO (Figure 5), we may see slight differences among fish samples. The lowest drying rate was observed in case of Nile tilapia followed by walking catfish.

**Figure 6:**
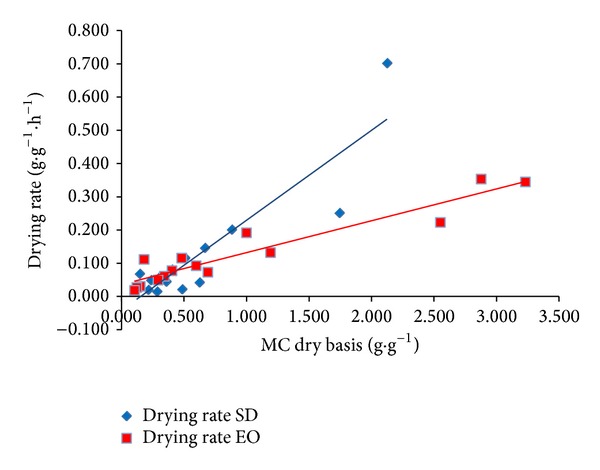
Drying rate curves of fish meat dried in solar dryer and electric oven.

Limited information is available on the kinetics of water removal from fish especially from species investigated in this study. Drying rates plotted with moisture contents for solar drying and EO drying are presented in Figure 6. The drying rates were higher at the beginning of the drying process and later decreased with decreasing moisture content. Similarly, as in case of Figures 4 and 5, a higher drying rate was observed during solar drying of fish mainly in the initial stages. The drying rates were fitted by linear trend lines and DR equations (11) and (12) were developed of solar drying and EO drying, respectively:

(11) $$\begin{array}{c} \text{DR}=0.0958\left(M\right)+0.0364\;\;\left(R^{2}=0.8994\right), \end{array}$$

(12) $$\begin{array}{c} \text{DR}=0.2703\left(M\right)-0.0406\;\;\left(R^{2}=0.8111\right). \end{array}$$

During the drying process, variations of DRs were observed which are caused by different shape, size, and nature of selected fish species. Similar results were reported by Jain and Pathare [36].

### 3.2. Mathematical Modeling of Drying Curves

**Figure 7:**
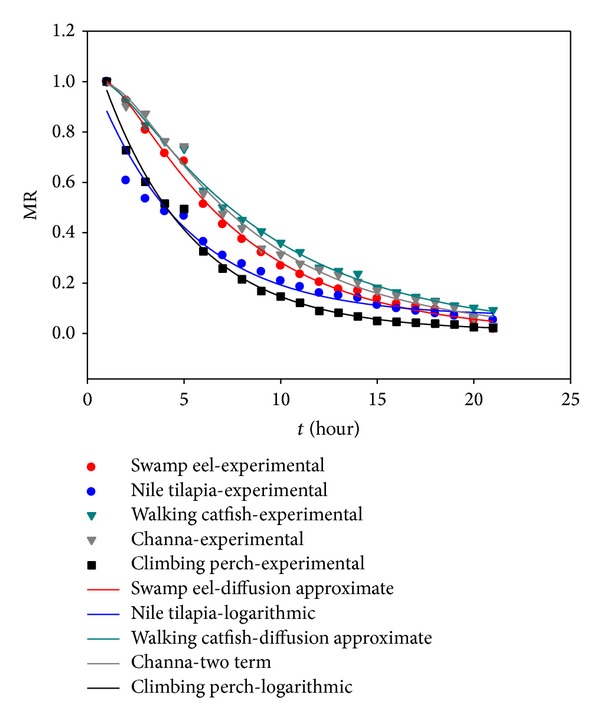
Experimental and predicted moisture ratio for solar drying of selected fish species.

**Figure 8:**
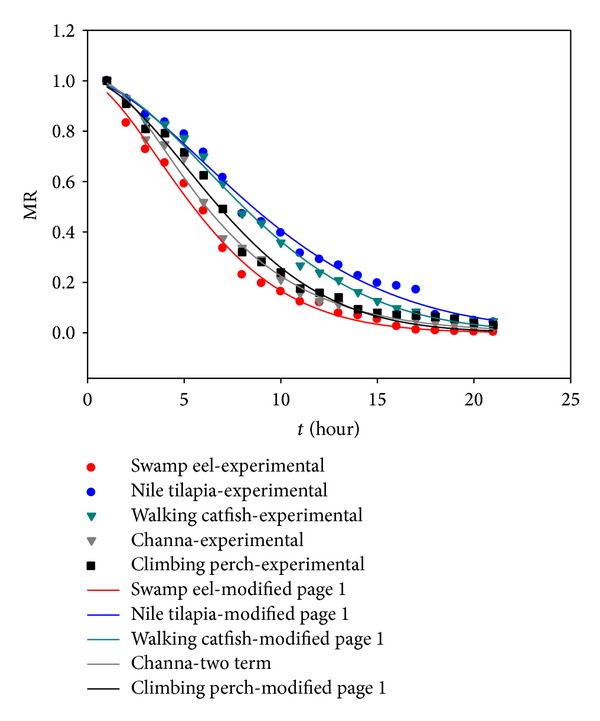
Experimental and predicted moisture ratio for EO drying of selected fish species.

The experimental data of moisture ratio versus drying time were fitted with ten drying models. The acceptability of the drying models was performed by correlation analyses, reduced chi-square (*χ* ^2^) test, and root-mean-square error (RMSE). Except for *χ* ^2^ and RMSE, the coefficient of determination (*R* ^2^) was used as the primary criterion to select the best equation to describe the drying curve as proposed by Erbay and Icier [37]. The results of statistical analyses are given in Table 2 for both solar and oven drying. As may be seen in case of solar drying, the logarithmic model, diffusion approximate model, and two-term model show the best suitability in describing the drying kinetics of climbing perch and Nile tilapia fish, swamp eel, walking catfish, and Channa fish, respectively. In case of drying in EO, the situation was more uniform since the Modified Page 1 model shows the best results for all investigated fish species except Channa fish where the two-term model may be considered as the most suitable one. Figures 7 and 8 show the variations of experimental and predicted moisture ratio values in case of solar and oven drying, respectively. In both figures, only the most suitable models with highest *R* ^2^ and lowest *χ* ^2^ and RMSE are presented. The values of *R* ^2^, *χ* ^2^, and RMSE for selected models in Figures 7 and 8 ranged from 0.964 to 0.997, 0.0008 to 0.0239, and 0.00036 to 0.00221, respectively. All the models gave better fits for oven drying than for solar drying, which is due to more uniform drying conditions in EO. In case of selected models, the *R* ^2^ values were greater than 0.96 indicating a good fit. Considering the uniform drying conditions and *R* ^2^, chi-square, and RMSE values for oven thin-layer drying, the Modified Page 1 model shows the best results. This may be due to the fact that the Modified Page 1 model is an empirical modification and has corrected the shortcomings of other theoretical and semitheoretical models considered. Similar observations for Page model were reported by Tunde-Akintunde [27]. As mentioned above, the situation for solar drying is quite different. In this case, two drying models, the logarithmic and two-term models, may be considered as the best to describe the drying kinetics of selected fish species. Jain and Pathare [36] also reported the logarithmic model as most suitable for solar drying of fish.

### 3.3. Organoleptic Properties and Sensory Analysis

**Figure 9:**
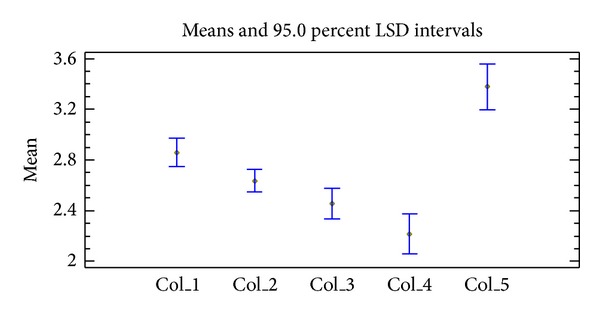
Evaluation of sensory analyses of dried fish samples. Col_1: Channa, Col_2: Nile tilapia, Col_3: walking catfish, Col_4: climbing perch, and Col_5: swamp eel.

Sensory data were analyzed using ANOVA and Fisher's LSD test. Statistically significant differences among fish species (5% confidence level) were determined. As shown in Figure 9, the best score in all tested categories (appearance, odour, flavor, texture, and overall sensory quality) was obtained in case of climbing perch, followed by Nile tilapia and walking catfish. Conversely, the worst scores were observed in case of Channa fish and swamp eel. As the result of the degustation panel, the dried meat from climbing perch fish was considered as the best in odour, flavor, and overall sensory quality. On the other hand, the dried meat from Nile tilapia had the best appearance and texture. There were no significant differences among meat samples dried in SD and EO.

## 4. Conclusions

Five most typical Cambodian fish species were selected for solar drying experiments in this study. Drying temperature and drying air relative humidity in the solar dryer were in average about 55.6°C and 19.9%, respectively. The overall solar dryer efficiency corresponding to 15% of final product moisture content was 12.37%. This is well in the typical range for natural convection solar dryers. An average evaporative capacity of solar dryer is 0.049 kg*·*h^−1^. Comparing the drying process in the solar dryer and control drying in electric oven, we may conclude that, in general, the drying rates were higher during solar drying. The drying rate equations for typical drying runs were developed for SD and EO drying. The drying curves from EO drying under constant conditions show slight differences among dried fish species. The lowest drying rate was observed in case of Nile tilapia followed by walking catfish. This is due to the structure of meat. Based on coefficient of determination (*R* ^2^), chi-square (*χ* ^2^) test, and root-mean-square error (RMSE), the most suitable mathematical models were selected. In case of natural convection solar drying, the most suitable models describing the drying kinetics were as follows: logarithmic model for climbing perch and Nile tilapia fish, the diffusion approximate model for swamp eel and walking catfish, and two-term model for Channa fish. Considering the uniform drying conditions in EO, the most appropriate mathematical model for all fish species is Modified Page 1 except for Channa fish where the two-term model shows better results. The results from the sensory evaluation of the dried fish samples show that most preferable fish is climbing perch, followed by Nile tilapia and walking catfish. There were no significant differences among meat samples dried in SD and EO in terms of the product quality. Finally, we may conclude that our study confirms solar drying as acceptable technology for fish processing in Cambodia and brings new knowledge about drying kinetics of locally typical fresh water fish species.

## Figures and Tables

**Table 1:** Mathematical models used to describe the drying characteristic of fish samples.

Model name	Models
Page	MR = exp⁡(−*k*t^*n*^)
Modified Page 1	MR = exp⁡[−(*kt*)^*n*^]
Modified Page 2	MR = exp⁡[(−*kt*)^*n*^]
Two-term exponential	MR = *a*exp⁡(−*k*t) + (1 − *a*)exp⁡(−*ka*t)
Diffusion approximate	MR = *a*exp⁡(−*k*t) + (1 − *a*)exp⁡(−*kb*t)
Thompson	*t* = *a*ln⁡MR + *b*(ln⁡MR⁡)^2^
Logarithmic	MR = *a*exp⁡(−*k*t) + *c*
Newton	MR = exp⁡(−*k*t)
Henderson and Pabis	MR = *a*exp⁡(−*k*t)
Two-term	MR = *a*exp⁡(−*k*_0_ *t*) + *c*exp⁡(−*k*_1_t)⁡

**Table 2:** Curve fitting criteria for various mathematical models and selected fish species during solar and oven drying.

Fish	Model name	*R* ^2^	RMSE	*χ* ^2^	Constants			
Solar dryer								

C. Perch	Page	0.9848	0.03285	0.00119	*k* = 0.1191	*n* = 1.2104		
	M. Page 1	0.9848	0.00216	0.00119	*k* = 0.1724	*n* = 1.2104		
	M. Page 2	0.9726	0.04411	0.00215	*k* = 0.1772	*n* = 1.0000		
	TT Ex.	0.985	0.03266	0.00118	*a* = 1.8083	*k* = 0.2497		
	D App.	0.9916	0.02438	0.00069	*a* = 1.1836	*k* = 0.2087	*b* = 2013.6754	
	Thompson	0.082	0.2551	0.07193	*a* = 0.5975	*b* = −0.2094		
	**L** **o** **g**.*	**0.9919**	**0.02396**	**0.00067**	**a** = 1.1865	**k** = 0.2160	**c** = 0.0100	
	Newton	0.9726	0.04411	0.00204	*k* = 0.1772			
	H. and P.	0.9916	0.02438	0.00066	*a* = 1.1836	*k* = 0.2087		
	T. Term.	0.9919	0.02395	0.00071	*a* = 1.1913	*b* = 0.0015	*k*_0_ = 0.2126	*k*_1_ = −0.0968

Channa	Page	0.9855	0.0355	0.00139	*k* = 0.0432	*n* = 1.3927		
	M. Page 1	0.9855	0.00252	0.00139	*k* = 0.1048	*n* = 1.3927		
	M. Page 2	0.947	0.06789	0.00509	*k* = 0.1059	*n* = 1.0000		
	TT Ex.	0.9894	0.03032	0.00102	*a* = 2.0122	*k* = 0.1704		
	D App.	0.9913	0.02748	0.00088	*a* = 0.4163	*k* = 0.6231	*b* = 0.2337	
	Thompson	0.2207	0.2603	0.07489	*a* = 0.9091	*b* = −0.3091		
	Log.	0.987	0.03357	0.00131	*a* = 1.2417	*k* = 0.1111	*c* = −0.0671	
	Newton	0.947	0.06789	0.00484	*k* = 0.1059			
	H. and P.	0.9849	0.03625	0.00145	*a* = 1.2094	*k* = 0.1289		
	**T. ** **T** **e** **r** **m**.*	**0.9915**	**0.02722**	**0.00092**	**a** = 1.3973	**b** = −0.4637	**k**_0_ = 0.1447	**k**_1_ = 0.7446

N. tilapia	Page	0.9598	0.04626	0.00237	*k* = 0.1964	*n* = 0.8978		
	M. Page 1	0.9598	0.00428	0.00237	*k* = 0.1632	*n* = 0.8978		
	M. Page 2	0.9538	0.04958	0.00272	*k* = 0.1600	*n* = 1.0000		
	TT Ex.	0.963	0.04441	0.00218	*a* = 0.3629	*k* = 0.3230		
	D App.	0.9632	0.04429	0.00229	*a* = 0.5267	*k* = 0.2682	*b* = 0.3737	
	Thompson	0.0566	0.23716	0.06216	*a* = 0.5785	*b* = −0.1953		
	**L** **o** **g**.*	**0.9645**	**0.04347**	**0.00221**	**a** = 1.0045	**k** = 0.2085	**c** = 0.0680	
	Newton	0.9538	0.04958	0.00258	*k* = 0.1600			
	H. and P.	0.954	0.04945	0.0027	*a* = 0.9845	*k* = 0.1573		
	T. Term.	0.954	0.04945	0.00302	*a* = 0.9845	*b* = 0.1252	*k*_0_ = 0.1573	*k*_1_ = 20.8265

S. eel	Page	0.9901	0.02947	0.00096	*k* = 0.0500	*n* = 1.3887		
	M. Page 1	0.9901	0.00174	0.00096	*k* = 0.1157	*n* = 1.3887		
	M. Page 2	0.9535	0.06394	0.00452	*k* = 0.1181	*n* = 1.0000		
	TT Ex.	0.9938	0.02328	0.0006	*a* = 2.0138	*k* = 0.1883		
	**D ** **A** **p** **p**.*	**0.9957**	**0.01939**	**0.00044**	**a** = −0.4022	**k** = 0.7316	**b** = 0.2189	
	Thompson	0.2114	0.26329	0.07662	*a* = 0.8566	*b* = −0.2943		
	Log.	0.9928	0.02517	0.00074	*a* = 1.2387	*k* = 0.1294	*c* = −0.0450	
	Newton	0.9535	0.06394	0.00429	*k* = 0.1181			
	H. and P.	0.9913	0.02764	0.00084	*a* = 1.2230	*k* = 0.1444		
	T. Term.	0.9957	0.01937	0.00046	*a* = 1.3934	*b* = −0.4156	*k*_0_ = 0.1596	*k*_1_ = 0.7856

W. catfish	Page	0.9919	0.02553	0.00072	*k* = 0.0468	*n* = 1.3255		
	M. Page 1	0.9919	0.0013	0.00072	*k* = 0.0992	*n* = 1.3255		
	M. Page 2	0.9621	0.05511	0.00336	*k* = 0.0996	*n* = 1.0000		
	TT Ex.	0.9936	0.02256	0.00056	*a* = 1.9306	*k* = 0.1542		
	**D ** **A** **p** **p**.*	**0.9961**	**0.01768**	**0.00036**	**a** = −0.2896	**k** = 0.7195	**b** = 0.1780	
	Thompson	0.1771	0.25666	0.07281	*a* = 0.9112	*b* = −0.3063		
	Log.	0.9941	0.02168	0.00055	*a* = 1.2032	*k* = 0.1058	*c* = −0.0515	
	Newton	0.9621	0.05511	0.00319	*k* = 0.0996			
	H. and P.	0.993	0.02363	0.00062	*a* = 1.1760	*k* = 0.1185		
	T. Term.	0.9961	0.01765	0.00038	*a* = −0.2788	*b* = 1.2960	*k*_0_ = 0.6639	*k*_1_ = 0.1284

Electric Oven								

C. Perch	Page	0.9886	0.03427	0.0013	*k* = 0.0249	*n* = 1.7353		
	**M. Page **1*	**0.9886**	**0.00235**	**0.0013**	**k** = 0.1190	**n** = 1.7353		
	M. Page 2	0.9137	0.09427	0.00982	*k* = 0.1227	*n* = 1.0000		
	TT Ex.	0.9873	0.03624	0.00145	*a* = 2.1650	*k* = 0.2073		
	D App.	0.9659	0.05924	0.00409	*a* = 1.2860	*k* = 0.1555	*b* = 160.1581	
	Thompson	0.2947	0.26956	0.08031	*a* = 0.8934	*b* = −0.3117		
	Log.	0.9742	0.05151	0.0031	*a* = 1.3314	*k* = 0.1226	*c* = −0.1082	
	Newton	0.9137	0.09427	0.00933	*k* = 0.1227			
	H. and P.	0.9659	0.05924	0.00388	*a* = 1.2860	*k* = 0.1555		
	T. Term.	0.9659	0.05924	0.00434	*a* = 1.6780	*b* = 1.2860	*k*_0_ = 65.5816	*k*_1_ = 0.1555

Channa	Page	0.9905	0.03073	0.00104	*k* = 0.0341	*n* = 1.6372		
	M. Page 1	0.9905	0.00189	0.00104	*k* = 0.1270	*n* = 1.6372		
	M. Page 2	0.927	0.08534	0.00805	*k* = 0.1318	*n* = 1.0000		
	TT Ex.	0.9919	0.02836	0.00089	*a* = 2.1452	*k* = 0.2201		
	D App.	0.9924	0.0275	0.00088	*a* = −1.4584	*k* = 0.2990	*b* = 0.9921	
	Thompson	0.2633	0.27114	0.08125	*a* = 0.8425	*b* = −0.2952		
	Log.	0.9831	0.04106	0.00197	*a* = 1.3130	*k* = 0.1388	*c* = −0.0782	
	Newton	0.927	0.08534	0.00765	*k* = 0.1318			
	H. and P.	0.9773	0.04759	0.0025	*a* = 1.2892	*k* = 0.1671		
	**T. ** **T** **e** **r** **m**.*	**0.9925**	**0.00273**	**0.00093**	**a** = −3.8854	**b** = 4.8618	**k**_0_ = 0.3499	**k**_1_ = 0.2654

N. tilapia	Page	0.9914	0.02875	0.00091	*k* = 0.0211	*n* = 1.6298		
	**M. Page **1*	**0.9914**	**0.00165**	**0.00091**	**k** = 0.0936	**n** = 1.6298		
	M. Page 2	0.9177	0.08904	0.00876	*k* = 0.0938	*n* = 1.0000		
	TT Ex.	0.9902	0.03069	0.00104	*a* = 2.1118	*k* = 0.1592		
	D App.	0.9729	0.05112	0.00305	*a* = −124.9620	*k* = 0.0176	*b* = 1.0219	
	Thompson	0.347	0.25084	0.06955	*a* = 1.0244	*b* = −0.3485		
	Log.	0.987	0.03534	0.00146	*a* = 1.4963	*k* = 0.0662	*c* = −0.3568	
	Newton	0.9177	0.08904	0.00832	*k* = 0.0938			
	H. and P.	0.9678	0.05567	0.00343	*a* = 1.2360	*k* = 0.1169		
	T. Term.	0.9678	0.05567	0.00383	*a* = 0.7523	*b* = 1.2360	*k*_0_ = 47.1655	*k*_1_ = 0.1169

S. eel	Page	0.9929	0.02588	0.00074	*k* = 0.0482	*n* = 1.5698		
	**M. Page **1*	**0.9929**	**0.00134**	**0.00074**	**k** = 0.1450	**n** = 1.5698		
	M. Page 2	0.9395	0.07532	0.00627	*k* = 0.1529	*n* = 1.0000		
	TT Ex.	0.9911	0.02882	0.00092	*a* = 2.0804	*k* = 0.2445		
	D App.	0.98	0.04326	0.00218	*a* = 1.2726	*k* = 0.1903	*b* = 136.9900	
	Thompson	0.2467	0.26579	0.07808	*a* = 0.7530	*b* = −0.2672		
	Log.	0.9873	0.03447	0.00139	*a* = 1.2849	*k* = 0.1579	*c* = −0.0716	
	Newton	0.9395	0.07532	0.00596	*k* = 0.1529			
	H. and P.	0.98	0.04326	0.00207	*a* = 1.2726	*k* = 0.1903		
	T. Term.	0.98	0.04326	0.00231	*a* = 1.2726	*b* = −1.0984	*k*_0_ = 0.1903	*k*_1_ = 22.4420

W. catfish	Page	0.996	0.02032	0.00046	*k* = 0.0179	*n* = 1.7527		
	**M. Page **1*	**0.996**	**0.00083**	**0.00046**	**k** = 0.1007	**n** = 1.7527		
	M. Page 2	0.91	0.09626	0.01024	*k* = 0.1024	*n* = 1.0000		
	TT Ex.	0.9905	0.03128	0.00108	*a* = 2.1509	*k* = 0.1744		
	D App.	0.9656	0.05951	0.00413	*a* = 523.1597	*k* = 0.0233	*b* = 0.9959	
	Thompson	0.3693	0.25487	0.0718	*a* = 1.0044	*b* = −0.3459		
	Log.	0.9835	0.04122	0.00198	*a* = 1.4536	*k* = 0.0785	*c* = −0.2882	
	Newton	0.91	0.09626	0.00973	*k* = 0.1024			
	H. and P.	0.9633	0.06145	0.00417	*a* = 1.2612	*k* = 0.1289		
	T. Term.	0.9633	0.06145	0.00466	*a* = 1.2612	*b* = −4.2422	*k*_0_ = 0.1289	*k*_1_ = 30.2247

∗Most appropriate mathematical model, M. Page 1: Modified Page 1, M. Page 2: Modified Page 2, TT Ex.: Two term exponential, D App.: Diffusion approximate, Log.: Logarithmic, H. and P.: Henderson and Pabis, T. Term.: Two term; C. Perch: Climbing perch, N. tilapia: Nile tilapia, S. eel: Swamp eel, W. catfish: Walking catfish.

## Nomenclature

*m*: Mass flow rate, kg*·*s^−1^
*C*: Specific heat of air, J*·*kg^−1°^C^−1^
*A*_*C*_: Collector area, m^2^
*T*_0_: Outlet air temperature, °C
*T*_*i*_: Inlet air temperature, °C
*I*: Global solar radiation on the plane of the collector, W*·*m^−2^
*W*: Mass of water removed from a wet product, kg
*L*: Latent heat of vaporization of water, J*·*kg^−1^
*m*_0_: Initial total crop mass, kg
*M*_*i*_: Initial moisture content fraction on wet basis
*M*_*f*_: Final moisture content fraction on wet basis
DR: Drying rate, kg*·*kg^−1^ *·*h^−1^
*t*: Drying time, s
MR: Moisture ratio
*M*: Moisture content of the materials at any time, kg*·*kg^−1^
*M*_0_: Initial moisture content on dry basis, kg
*M*_*e*_: Equilibrium moisture content on dry basis, kg
MR_exp⁡,*i*_: Experimental value of moisture ratio
MR_pre,*i*_: Predicted value of moisture ratio
*N*: Number of observations
*z*: Number of constants in drying model
*L*: Half-thickness of the samples, m
*E*: Evaporative capacity, kg*·*h^−1^
*X*_2*m*_: Dryer outlet absolute humidity
*X*_*a*_: Ambient absolute humidity
*a*, *b*, *c*, *g*, *k*, *n*: Constants.

### Greek Symbols

Δ*W*: Weight loss in one hour interval, kg*·*kg^−1^
Δ*T*: Difference in time reading, h
*η*_*c*_: Thermal efficiency of a solar collector, %
*η*_*p*_: System drying efficiency.
